# Supplementary material for: The Effect of the ZrO2 Loading in SiO2@ZrO2-CaO Catalysts for Transesterification Reaction
Source: Materials (Basel). 2020 Jan 4;13(1):221. doi: 10.3390/ma13010221 (PMC6981678; doi:10.3390/ma13010221)
Supplement: Supplementary file 1 [file materials-13-00221-s001.pdf]

materials

Supplementary Materials

The Effect of the ZrO_2 Loading in SiO_2@ZrO_2-CaO Catalysts for Transesterification Reaction

Daniela Salinas [1]*, Sichem Guerrero [2], Cristian H. Campos [3], Tatiana M. Bustamante [3] and Gina Pecchi [3,4]

1 Departamento de Química, Universidad del Biobío, Avenida Collao 1202, 4030000, Concepción, Chile
2 Facultad de Ingeniería y Ciencias Aplicadas, Universidad de Los Andes, Monseñor Álvaro del Portillo 12455, Las Condes, 7550000, Santiago, Chile; sguerrero@uandes.cl
3 Departamento de Físico-Química, Facultad de Ciencias Químicas, Universidad de Concepción, Edmundo Larenas 129, 4030000, Concepción, Chile; ccampos@udec.cl (C.H.C.); tatibustamante@udec.cl (T.M.B.), gpecchi@udec.cl (G.P.)
4 Millenium Nuclei on Catalytic Processes towards Sustainable Chemistry (CSC), 8940000, Santiago, Chile
* Correspondence: dsalinas@ubiobio.cl

Received: 27 November 2019; Accepted: 2 January 2020; Published: 4 January 2020

Table 1. Surface area of the SiO_2@ZrO_2 core@shell without calcination (fresh samples).

Catalyst	BET Surface Area ($m^2 g^{-1}$)
SiO_2 spheres	17
SiO_2@ZrO_2 0.04M	153
SiO_2@ZrO_2 0.06M	192
SiO_2@ZrO_2 0.08M	150
SiO_2@$ZrO_2$0.04M-CaO	n.d
SiO_2@$ZrO_2$0.06M-CaO	n.d
SiO_2@$ZrO_2$0.08M-CaO	n.d

Table 2. Fatty acid composition of the canola oil.

Fatty Acid Composition	Composition of Canola Oil, wt %
Palmitic (C16:0)	4.4
Oleic (C18:1)	55
Linoleic (C18:2)	31
Linolenic (C18:3)	7.8

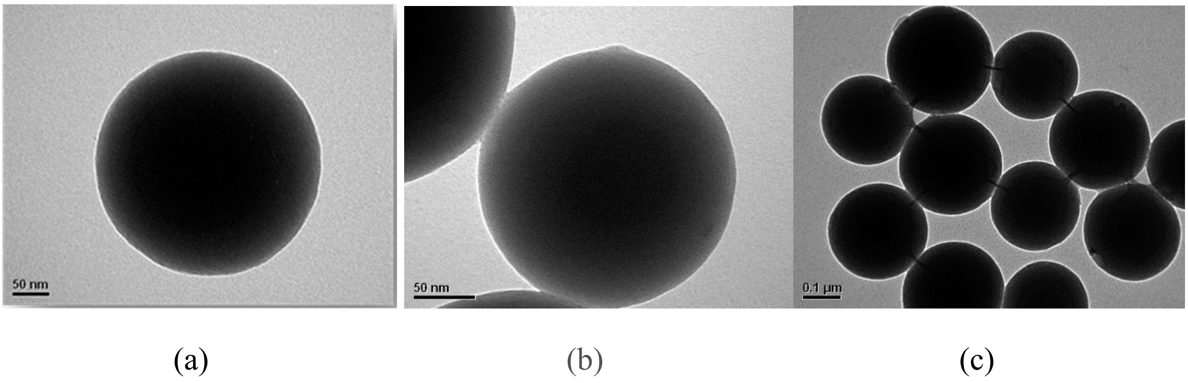

Figure 1. TEM images (**a**) SiO_2 sphere; (**b**) SiO_2 sphere—500°C; (**c**) SiO_2 sphere—700°C.

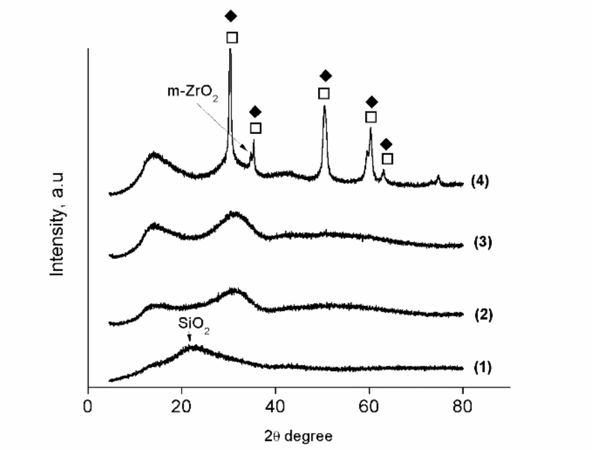

Figure 2. XRD profiles of SiO_2 sphere (1) and core@shell SiO_2@ZrO_2 0.04M (2), SiO_2@ZrO_2 0.06M (3) and SiO_2@ZrO_2 0.08M (4) calcined at 500 °C.

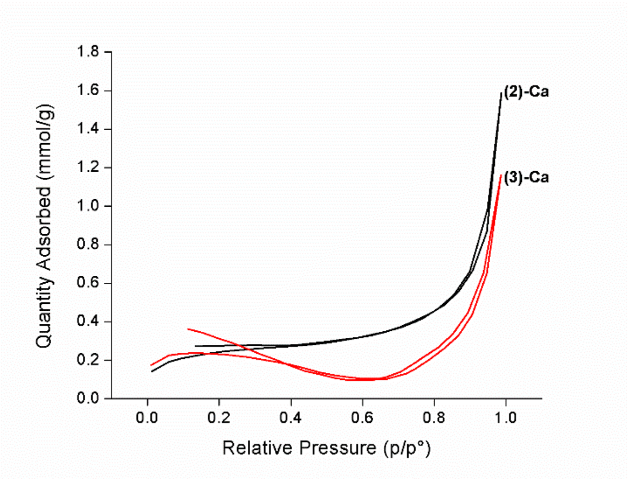

Figure 3. N_2 adsorption-desorption isotherms of (2)-Ca: SiO_2@ZrO_2 0.06M-CaO, (3)-Ca: SiO_2@ZrO_2 0.08M-CaO calcined at 700°C.

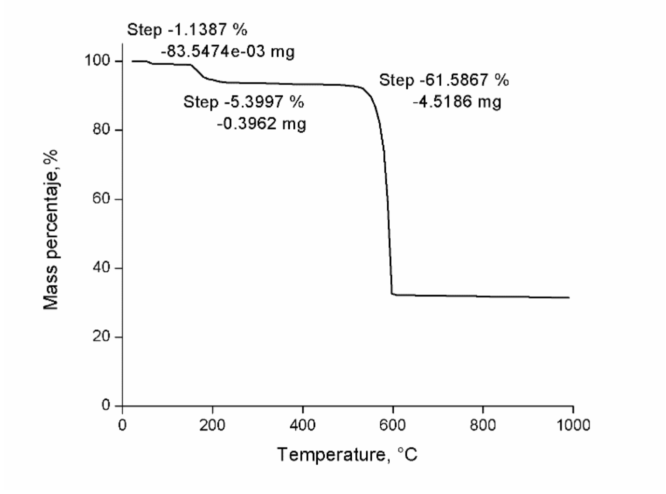

Figure 4. TGA of $CaNO_3{\cdot}4H_2O$.

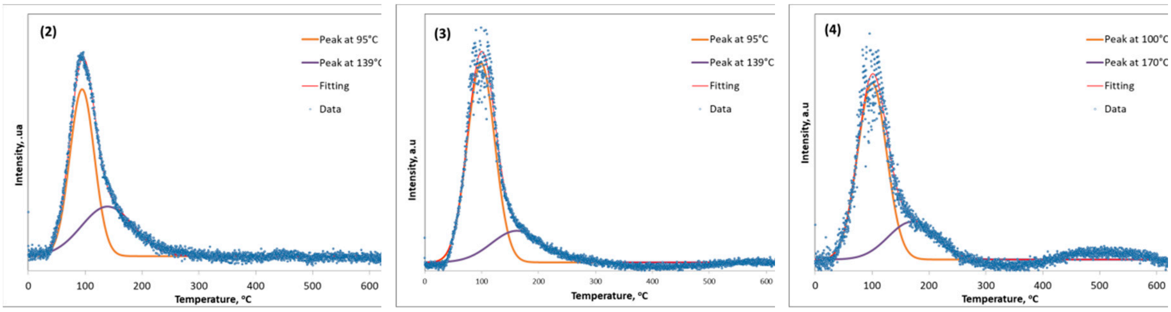

(a) **(b)** **(c)**

Figure 5. CO_2 temperature-programmed desorption profiles of $SiO_2@ZrO_2$ 0.04M (2), $SiO_2@ZrO_2$ 0.06M (3) and $SiO_2@ZrO_2$ 0.08M (4) at 700 °C. Software Fityk for deconvolution and Levenberg-Marquardt method for peak fitting, (**a**) $SiO_2@ZrO_2$ 0.04M; (**b**) $SiO_2@ZrO_2$ 0.06M and (**c**) $SiO_2@ZrO_2$ 0.08M.

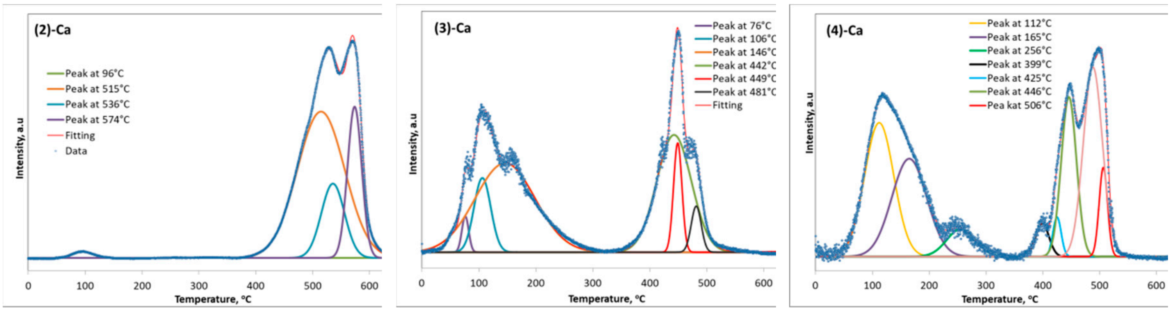

(a) **(b)** **(c)**

Figure 6. CO_2 temperature-programmed desorption profiles of $SiO_2@ZrO_2$ 0.04M-CaO (2)-Ca, $SiO_2@ZrO_2$ 0.06M-CaO (3)-Ca and $SiO_2@ZrO_2$ 0.08M-CaO (4)-Ca at 700 °C. Software Fityk for deconvolution and Levenberg-Marquardt method for peak fitting. (**a**) $SiO_2@ZrO_2$ 0.04M-CaO ; (**b**) $SiO_2@ZrO_2$ 0.06M-CaO and (**c**) $SiO_2@ZrO_2$ 0.08M-CaO.

© 2020 by the authors. Licensee MDPI, Basel, Switzerland. This article is an open access article distributed under the terms and conditions of the Creative Commons Attribution (CC BY) license (http://creativecommons.org/licenses/by/4.0/).
